# Thoracoscopically assisted resection of left ventricular myxoma: a case report

**DOI:** 10.3389/fcvm.2026.1798924

**Published:** 2026-05-20

**Authors:** Yuhui Zhang, Fenlong Xue, Mingzhen Qin, Junshan Li, Yanhe Ma, Lianqun Wang, Zhigang Guo

**Affiliations:** 1 Department of Cardiac Surgery, Tianjin Chest Hospital, Tianjin, China; 2 Department of Medical Imaging, Tianjin Chest Hospital, Tianjin, China

**Keywords:** cardiac tumor, case report, left ventricular myxoma, thoracoscopy, tumor resection

## Abstract

**Background:**

Cardiac myxoma is the most common primary benign cardiac tumor, with 95% occurring in the atria and only about 3% in the ventricles. Left ventricular (LV) myxomas are even rarer. These tumors can lead to adverse consequences, such as heart failure, arrhythmias, and even sudden death. Surgical resection is the preferred treatment, and complete resection is necessary to prevent late recurrence.

**Case presentation:**

We present the case of a 63-year-old female patient who was diagnosed with a LV myxoma by multimodal imaging and successfully underwent thoracoscopically assisted surgical resection with a good prognosis.

**Conclusions:**

This case discusses the clinical characteristics, as well as key points in the diagnosis and treatment, through the case of a patient with LV myxoma.

## 1 Introduction

Cardiac myxoma represents the most histologically benign primary tumor of the heart, accounting for 50% of all primary cardiac tumors. Its global prevalence is 0.03%, with an annual incidence ranging from 0.5 to 1 case per million individuals (1, 2). Most cases occur in individuals aged 40–60 years, and females are two to three times more likely to be affected than males. Approximately 95% of cardiac myxomas occur in the atrium, with only 2%–3% originating in the left ventricle (LV) (3, 4). Cardiac myxoma can be classified into two epidemiological forms: sporadic and familial. Approximately 5%–10% of myxoma cases show familial clustering, most commonly presenting as Carney Complex (CNC). CNC is an autosomal dominant genetic disorder caused by mutations in the *PRKAR1A* gene and characterized by myxoma, hyperpigmentation, endocrine hyperactivity (5).

The etiopathogenesis of cardiac myxomas remains incompletely understood. It has been hypothesized that these tumors from multipotent mesenchymal cells embedded in a myxoid stroma and are histologically composed of polygonal and stellate cells with hyperchromatic nuclei. Myxoma tissues are capable of secreting various cytokines and vascular endothelial growth factor, thereby promoting angiogenesis and tumor progression, as well as inducing the release of inflammatory mediators such as interleukin-6 (IL-6). Notably, IL-6 is considered a more sensitive indicator than C-reactive protein for reflecting the inflammatory status in patients with cardiac myxom (5, 6). In patients with CNC, myxomas are often multiple and frequently associated with mutations in the *PRKAR1A* gene, which lead to dysregulation in the cAMP/PKA signaling pathway and disruption of normal cellular function (7). In sporadic myxomas, recurrent somatic mutations have been identified in *PRKAR1A*, as well as in other genes involved in the cAMP pathway, such as *PRKACA* and *PDE11A*, suggesting that dysregulation of cAMP signaling is a common mechanism underlying both sporadic and familial myxomas (8).

Although cardiac myxomas are histologically benign, the resulting intracardiac masses may lead to serious consequences such as embolism, heart failure, and sudden death (9). Given its low incidence, LV myxoma is prone to being misdiagnosed as LV thrombus in clinical practice (10). This diagnostic challenge can delay timely management and consequently lead to fatal complications. Generally, surgery is the main treatment for cardiac myxomas (11), and the surgical approaches depend on the location of the intracardiac myxoma (12). Currently, transthoracic echocardiography (TTE) is commonly used to visualize the myxoma and its relationship with surrounding structures (13). Myocardial contrast echocardiography (MCE) has been used as an alternative because it can effectively assess LV myxoma perfusion (14). In complex cases, cardiac magnetic resonance (CMR) imaging can provide key diagnostic information through multi-sequence analysis (15). This multimodal evaluation is highly valuable for determining the nature of the tumor and treatment decisions (16). The multimodal imaging not only confirmed the diagnosis but also clarified the tumor's attachment and its spatial relationship with surrounding structures, which made video-assisted thoracoscopic surgery (VATS) a feasible and safe minimally invasive approach.

In this case report, we presented a rare case of LV myxoma diagnosed by multimodal image combination method, using echocardiographic examination, CMR and MCE, and successfully treated via VATS under cardiopulmonary bypass (CPB).

## 2 Case presentation

A 63-year-old female patient with a history of cerebral infarction and mass in the LV septum presented to our department for the regular physical examination. CMR imaging (Supplementary Video 1) identified an irregular intraventricular mass attached to the anterior aspect of the mid-to-distal ventricular septum, low T1WI signals, and high T2WI/SPAIR signals, which were consistent with the features of a myxoma. Cranial MRI revealed multiple ischemic infarctions involving the left cerebellar hemisphere, bilateral occipital lobes, corpus callosum, and right parietal lobe, with a subacute cerebral infarction in the left cerebellar hemisphere was considered. Thoracic CT angiography (CTA) (Supplementary Video 2) revealed mild stenosis in the middle-segment of the anterior descending coronary artery, without evidence of pulmonary embolism, or aortic dissection. A mass in the LV cavity was noted, raising suspicion for myxoma.

**Figure 1:**
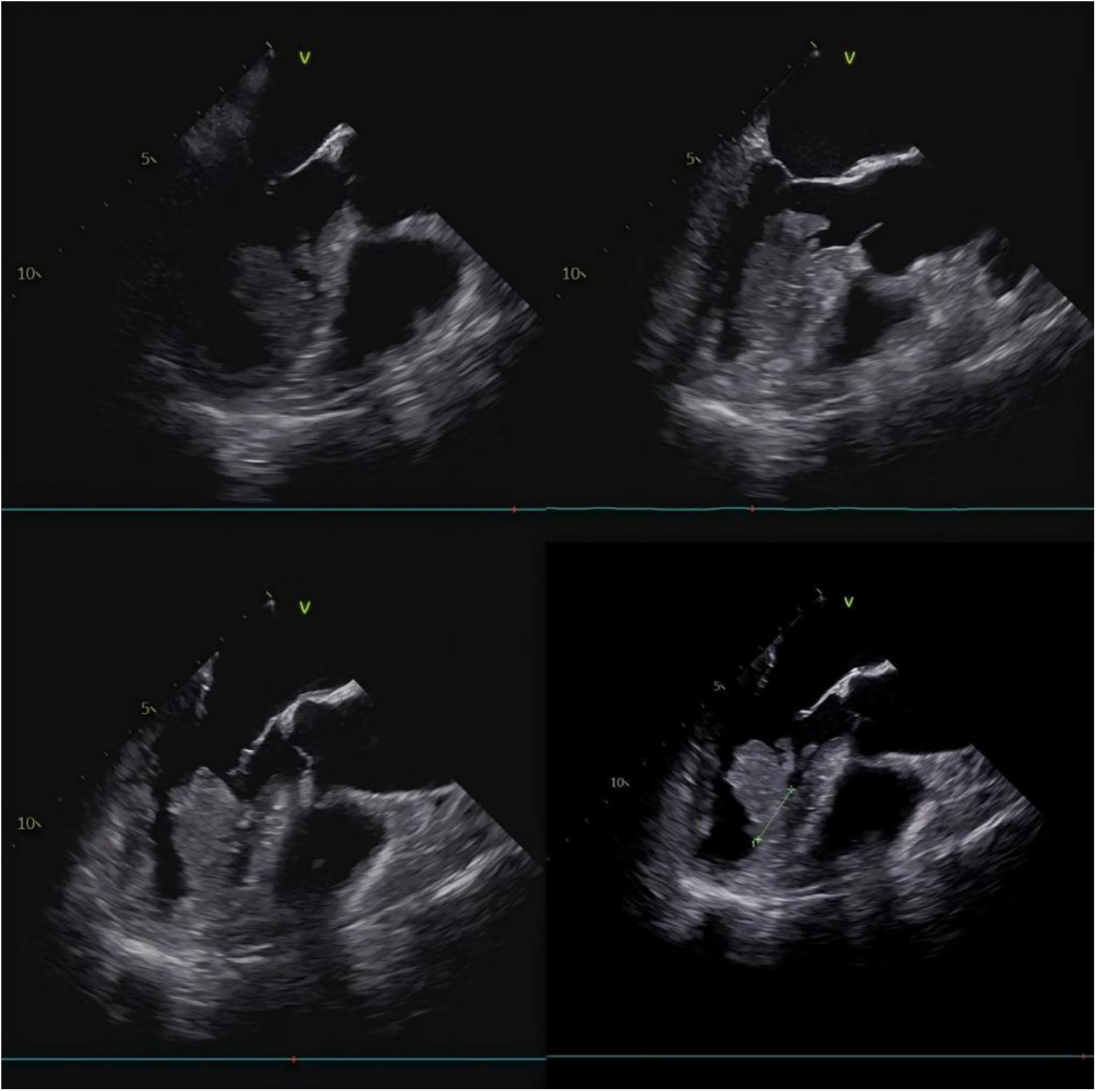
Preoperative transesophageal echocardiography demonstrating left ventricular cavity with multi-angle assessment of mass morphology, dimensions, and basal attachment.

After excluding surgical contraindications, cardiac tumor resection under CPB was planned based on the tumor's size, shape, texture, and site of pedicle attachment. The surgical approaches included: a transaortic incision via the aortic valve; a right atrial incision via the interatrial septum or atrioventricular groove to assess the mitral valve; and a LV incision. Intraoperative TEE was shown in the figure (Figure 1). We ultimately selected the aortic valve approach and employed VATS to enhance visualization. During this approach, the aortic valve leaflets should be handled gently to avoid causing aortic regurgitation. Particular caution is required when operating near the junction of the right coronary and non-coronary sinuses to prevent injury to the atrioventricular node, thereby avoiding severe atrioventricular conduction block.

**Figure 2:**
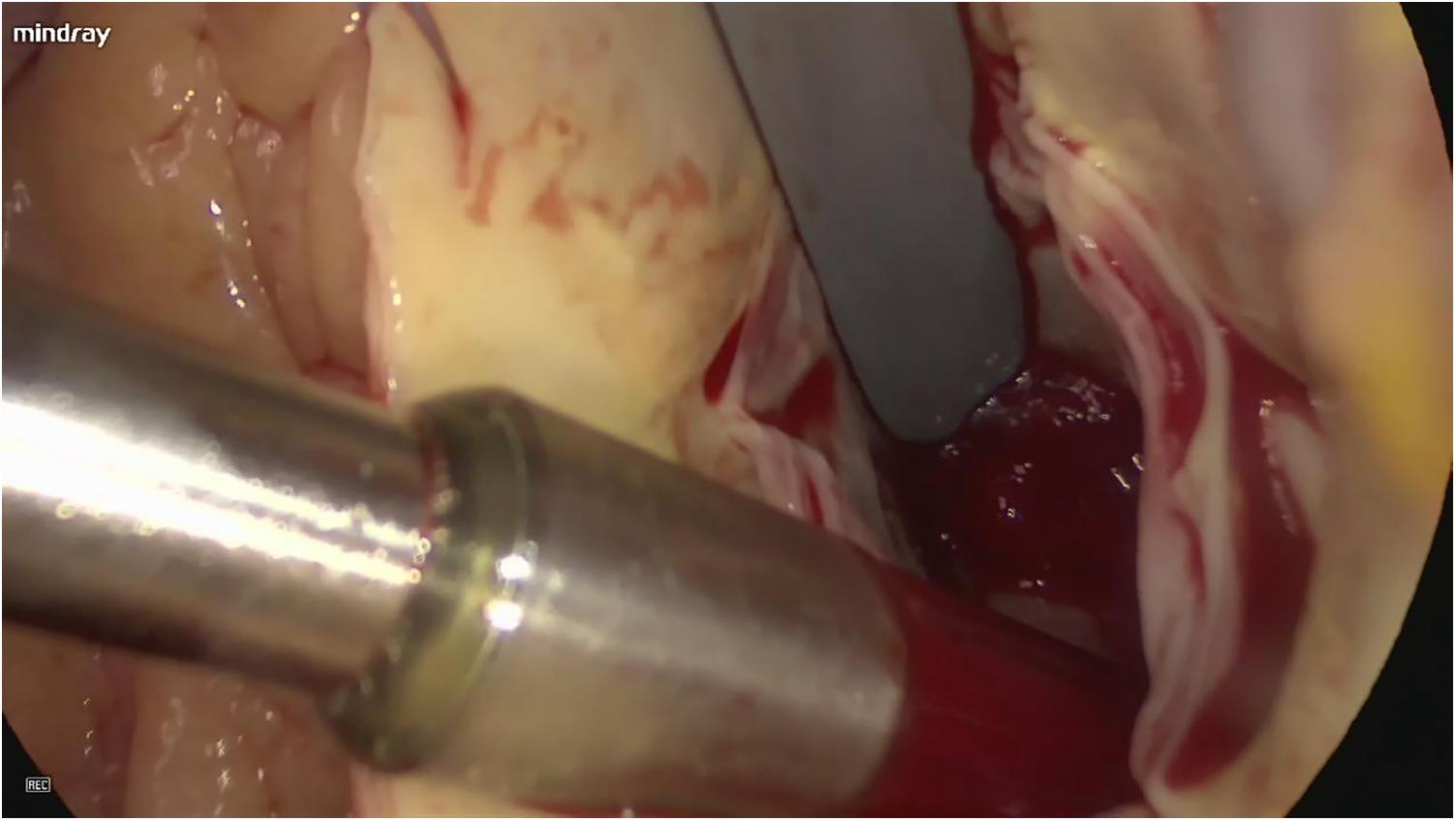
Intraoperative photograph of tumor with endocardial attachment pre-resection.

**Figure 3:**
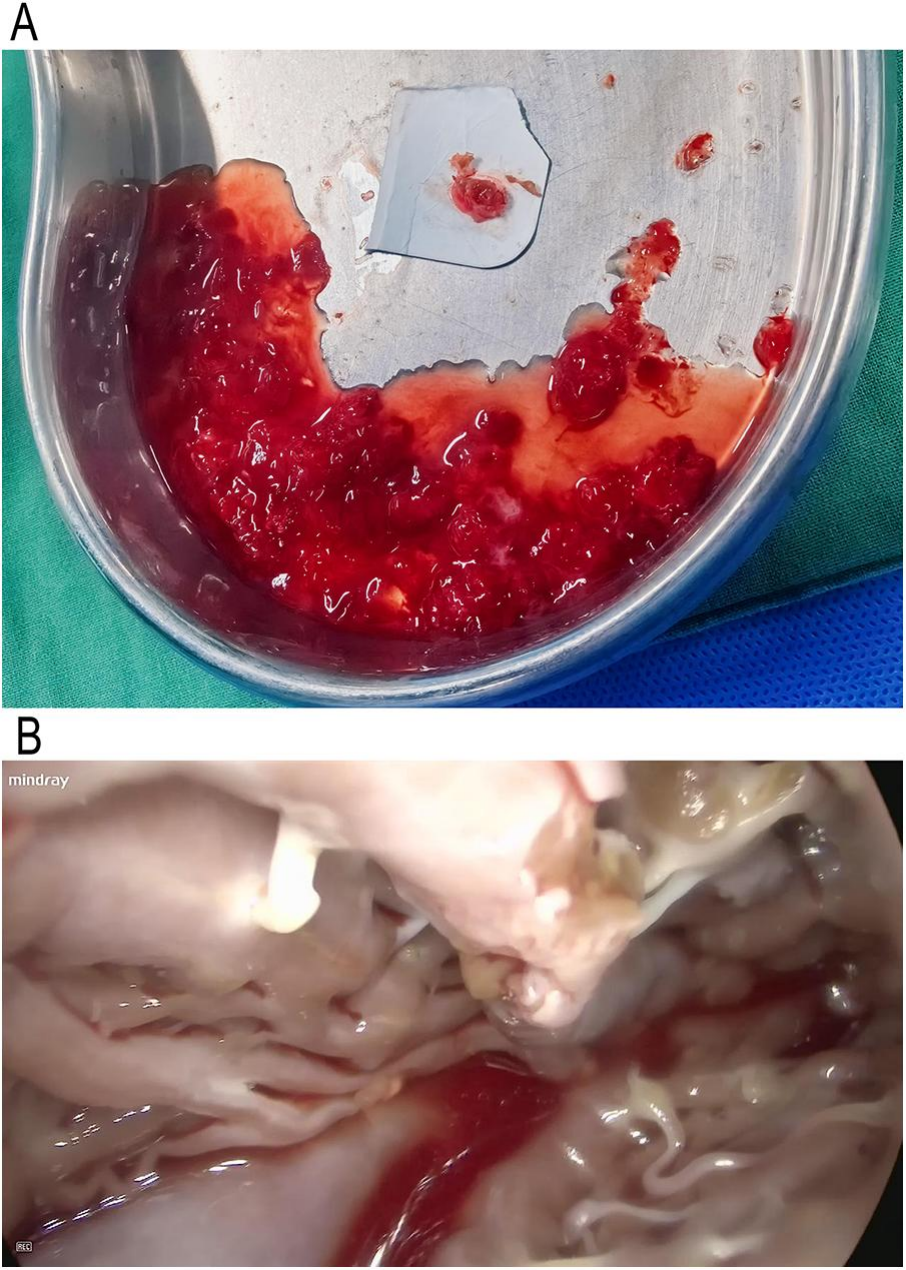
Macroscopic aspect of the tumoral mass **(A)** intraoperative photograph of tumor with endocardial attachment during resection **(B)**.

A median sternotomy was performed under general anesthesia. CPB was established via cannulation of the ascending aorta, superior and inferior vena cava. The activated clotting time (ACT) was maintained at > 480 s. CPB was then initiated. A del Nido perfusion tube was inserted into the aortic root, and systemic temperature was lowered to 32 °C. The ascending aorta was then cross-clamped, while the superior and inferior vena cava were not clamped. The aortic root was perfused with cardioplegia solution in an antegrade manner, and a LV vent was inserted through the right superior pulmonary vein. Topical ice slush was applied to the heart surface to assist myocardial cooling, and cardiac arrest was achieved. The ascending aorta was incised obliquely, and a jelly-like tumor was located below the right coronary sinus of the aorta (Figure 2). The tumor was removed via a thoracoscopically assisted approach. Upon exploration, the tumor pedicle was found to originate from the trabecular myocardium of the ventricular septum. Two pedicles were identified, arising from the endocardium overlying the trabecular myocardium of the ventricular septum. The tumor pedicle tissues were removed along with the surrounding endocardium and adjacent myocardium (Figures 3A, B, Supplementary Video 3).

**Figure 4:**
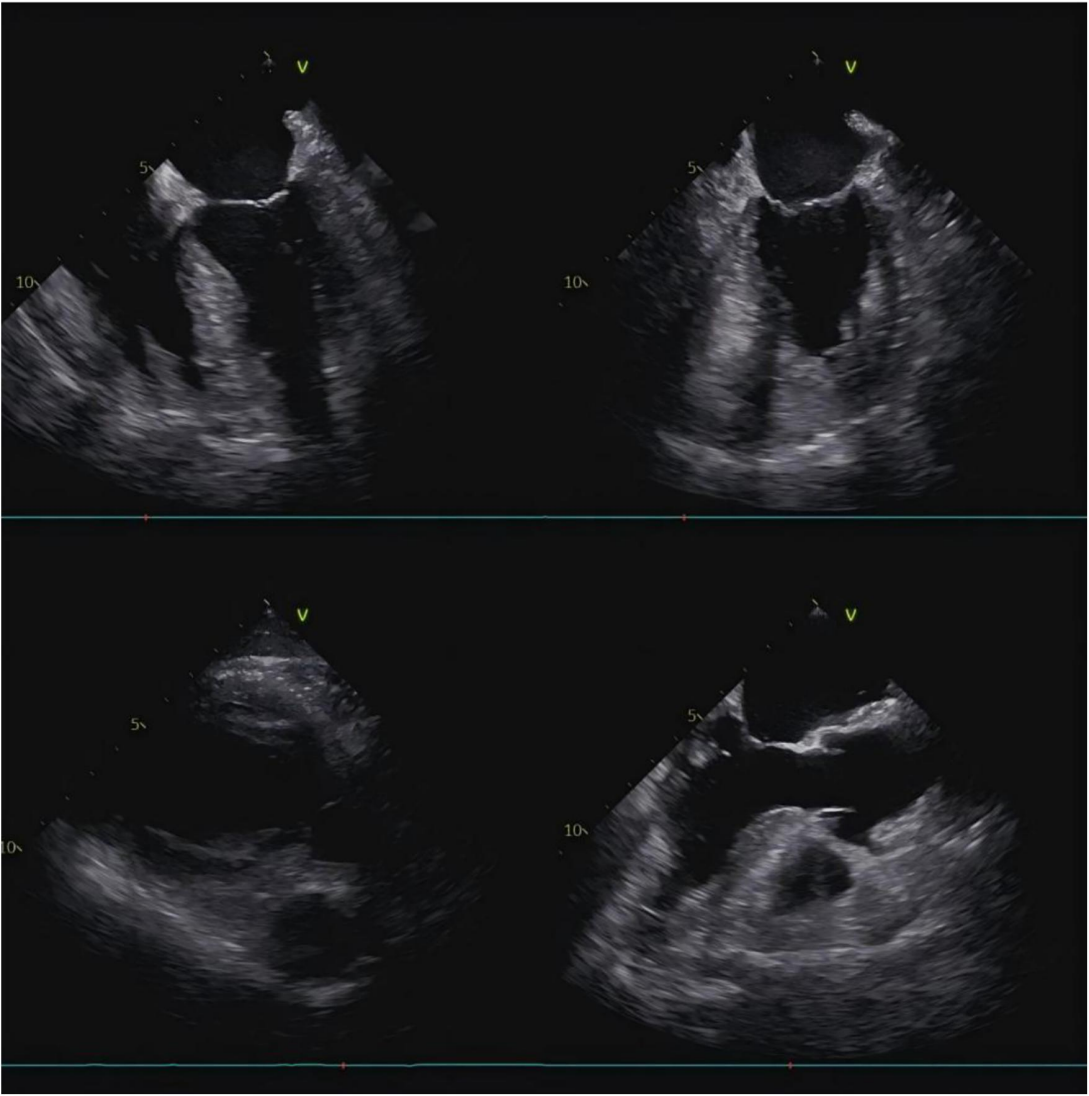
Postoperative transesophageal echocardiography showing no residual mass in left ventricular cavity from multiple angles.

The aortic incision was closed with continuous sutured. LV venting was initiated, and systemic rewarming was continued until the body temperature reached 36.5 °C. After volume adjustment and stabilizing circulation, CPB was stopped. Protamine was administered to neutralize heparin, followed by meticulous hemostasis and layered closure of the incision. Postoperative TEE examination demonstrated no regurgitation of the aorta or mitral valve, and no residual mass (Figure 4, Supplementary Video 4). Paroxysmal atrial fibrillation occurred postoperatively but was successfully converted to sinus rhythm after treatment. The resected tumor was sent to pathological examination, which confirmed the diagnosis cardiac myxoma (Figure 3A). The patient was recovered well, experience no postoperative complications, and discharged uneventfully. At 3-month follow-up, the patient reported good recovery and remained free of tumor recurrence.

## 3 Discussion

Approximately 90% of cardiac myxoma are sporadic, while about 5%–10% are familial (17). Our case was classified as sporadic as the patient did not exhibit familial clustering characteristics. Approximately 80% of cardiac myxomas occur in the left atrium, with the pedicle attached to the fossa ovalis of the atrial septum. About 15% originate in the right atrium, usually attaching to the right atrial wall or the atrial septum. The remaining 3%–4% arise in the ventricles, with attachments to the ventricular septum, free wall, or mitral valve chordae (18). In this study, we reported a rare case with cardiac tumor attaching to the LV septum.

The clinical manifestations of cardiac myxoma are usually nonspecific. Patients with cardiac myxoma may experience symptoms such as fatigue, low-grade fever, and weight loss (19). Their laboratory findings include chronic hemolytic anemia, polycythemia, leukocytosis, an accelerated erythrocyte sedimentation rate, and elevated levels of C-reactive protein and immunoglobulins (20). The main clinical manifestations are featured by alternations in the cardiac hemodynamics. Left atrial myxoma may present with symptoms similar to mitral stenosis, such as pulmonary congestion, pulmonary hypertension, postural dyspnea, and LV dysfunction (17). Tumors located in the right atrium may present with dyspnea, pulmonary embolism, systemic congestion, and right ventricular dysfunction (21). Tumors located in the ventricles may be associated with the risk of arrhythmia, systemic embolism, cerebral infarction, and even sudden death (22). In this case, the patient experienced a cerebral infarction 6 months ago, and a mass was found in the left ventricle, which was suspected to be a thrombus or myxoma. Considering that the ventricular tumor may be related to the cerebral infarction, and given that the LV mass increased significantly 6 months later, it was more likely diagnosed as a cardiac myxoma.

As cardiac myxomas are typically friable in texture, it is prone to fragmentation and embolization. Large tumors may result in heart failure, arrhythmias, hemodynamic disturbances, and even sudden death. Therefore, surgical resection is recommended upon diagnosis (23, 24). The surgical approach and resection strategy should be determined based on the tumor size and location (25). For tumors arising from the atrium, resection can be performed under general anesthesia with cardiopulmonary bypass via median sternotomy to remove the tumor through the right atrium and atrial septum (26). Alternatively, totally thoracoscopic surgical resection can be performed, showing good safety and efficacy (27). However, surgical resection may pose increased risk for patients in poor clinical condition. Therefore, radiofrequency ablation and embolization may be considered as alternative therapeutic options (6). Zheng et al. reported a patient with a right ventricular giant myxoma and outflow tract stenosis who was not a candidate for surgical resection but was successfully treated with radiofrequency ablation, resulting in symptom improvement (28). In addition, there are no pharmacological treatments for cardiac myxomas, and drugs therapy is primarily used to manage associated complications, such as heart failure or arrhythmias. Emerging molecular approaches including gene editing, RNA-based treatments, and immunotherapy, offer potential for complex or recurrent cases, but their clinical translation remains at an early stage (6).

To the best of our knowledge, LV myxomas are rare, with most of the publications consisting of case reports. For instance, Asad et al. reported a case of complete resection of a LV myxoma with a size of approximately 2 × 2 cm through an aortic incision and aortic valve (4). Greco et al. reported a LV myxoma with removal of tumor based on video-assisted cardioscopy (29). Yamada et al. described the removal of a left atrial myxoma through right thracotomy after Bentall surgery (30). Kudo et al. reported the removal of a LV tumor via trans-mitral endoscopic approach (31). In these cases, the tumors typically exhibited intact capsules and limited pedicles, making complete resection more feasible. In our case, the tumor showed a lobed structure, loose texture, and fragility. Single procedure for removal of the tumor is a challenge as the pedicle was located between the pectinate muscles of the ventricular septum, and the base was wide. On this basis, a thoracoscopically assisted approach was employed, providing exposure of the surgical field via an aortic incision across the aortic valve. Under the thoracoscopic field, the tumor pedicle was found, and the adjacent endocardium and part of the myocardium were completely excised to reduce the risk of recurrence. Although the patient showed left bundle branch block after surgery, it did not affect cardiac function, and the structures of the aortic and mitral valves remained intact. No embolic events or adverse complications occurred post-surgery, and no recurrence was observed during follow-up.

This approach was selected to minimize myocardial injury and protect cardiac function. Compared with conventional median sternotomy with open-heart surgery, the thoracoscopic-assisted approach allows enhanced visualization through a smaller incision while avoiding a direct LV incision, thereby reducing myocardial trauma. Compared with minimally invasive small-incision thoracoscopic surgery via the mitral valve, this approach avoids the challenge of the anterior mitral leaflet obstructing tumor excision, which in conventional thoracoscopic approaches may require temporary resection and reimplantation of the leaflet, potentially causing valvular injury. Therefore, the thoracoscopic-assisted median sternotomy provides a balanced approach that enables complete tumor removal, preserves myocardial and valvular integrity, and combines the advantages of minimally invasive visualization with the safety of direct access.

Minimally invasive cardiac procedures are performed through a smaller skin incision, often without or with only partial sternal division, leading to reduced surgical trauma (32). However, whether more minimally invasion approaches can be safely and effectively applied remains an important question. LV tumor are classified as benign and malignant. Benign tumor are typically clear boundaries and can be easily separated from surrounding structures, facilitating complex excision. In such cases, an appropriate surgical approach based on the location of the tumor pedicle may allow for minimally invasive resection, thereby reducing surgical trauma, promoting faster postoperative recovery, and achieving favorable clinical outcomes. In contrast, malignant tumors often exhibit infiltrative growth and close adherence to adjacent tissues, making complete resection more challenging and increasing the technical demands of minimally invasive surgery (33). Therefore, surgical strategies should be individualized based on tumor characteristics, including pathological nature, location, and resectability, as well as the surgeon's expertise. Minimally invasive approaches should be considered only when complete tumor excision can be ensured.

## 4 Conclusion

In summary, surgical resection can yield favorable outcomes for cardiac myxoma. It is crucial to select the appropriate surgical approach based on the tumor's location, size, and the surrounding cardiac structures. Thoracoscopic surgery aids in the complete removal of tumor tissue and pedicles, thereby reducing the risk of recurrence.

## Data Availability

The original contributions presented in the study are included in the article/supplementary material, further inquiries can be directed to the corresponding author.

## Supplementary material

SUPPLEMENTARY VIDEO 1 Cardiac examination image.

SUPPLEMENTARY VIDEO 2 Chest CTA image.

SUPPLEMENTARY VIDEO 3 Intraoperative thoracoscopic resection of left ventricular myxoma.

SUPPLEMENTARY VIDEO 4 Postoperative TEE examination images.
